# Above the Noise: The Search for Periodicities in the Inner Heliosphere

**DOI:** 10.1007/s11207-017-1191-3

**Published:** 2017-10-31

**Authors:** James Threlfall, Ineke De Moortel, Thomas Conlon

**Affiliations:** School of Mathematics and Statistics, Mathematical Institute, University of St Andrews, St Andrews, KY169SS UK

**Keywords:** Corona, active, Magnetic fields, corona, Oscillations, solar, Solar wind, theory

## Abstract

Remote sensing of coronal and heliospheric periodicities can provide vital insight into the local conditions and dynamics of the solar atmosphere. We seek to trace long (one hour or longer) periodic oscillatory signatures (previously identified above the limb in the corona by, *e.g.*, Telloni *et al.* in *Astrophys. J.* **767**, 138, 2013) from their origin at the solar surface out into the heliosphere. To do this, we combined on-disk measurements taken by the *Atmospheric Imaging Assembly* (AIA) onboard the *Solar Dynamics Observatory* (SDO) and concurrent extreme ultra-violet (EUV) and coronagraph data from one of the *Solar Terrestrial Relations Observatory* (STEREO) spacecraft to study the evolution of two active regions in the vicinity of an equatorial coronal hole over several days in early 2011. Fourier and wavelet analysis of signals were performed. Applying white-noise-based confidence levels to the power spectra associated with detrended intensity time series yields detections of oscillatory signatures with periods from 6 – 13 hours in both AIA and STEREO data. As was found by Telloni *et al.* (2013), these signatures are aligned with local magnetic structures. However, typical spectral power densities all vary substantially as a function of period, indicating spectra dominated by red (rather than white) noise. Contrary to the white-noise-based results, applying global confidence levels based on a generic background-noise model (allowing a combination of white noise, red noise, and transients following Auchère *et al.* in *Astrophys. J.* **825**, 110, 2016) without detrending the time series uncovers only sporadic, spatially uncorrelated evidence of periodic signatures in either instrument. Automating this method to individual pixels in the STEREO/COR coronagraph field of view is non-trivial. Efforts to identify and implement a more robust automatic background noise model fitting procedure are needed.

## Introduction

The solar corona hosts a variety of dynamic phenomena that generate periodic and aperiodic variations in light intensity. These signals are vital clues in our efforts to unlock the secrets of this enigmatic region of the Sun, providing insight into how dynamic phenomena contribute to both the coronal heating problem (*e.g.* De Moortel and Browning, 2015) and the acceleration of the solar wind (*e.g.* Abbo *et al.*, 2016, and references therein). It is now widely agreed that waves are ubiquitous throughout the solar corona (De Moortel and Nakariakov, 2012). The number and diversity of observed structures/locations that support oscillatory signatures are incredibly wide-ranging. Signatures have also been inferred across an enormous range of timescales, from the longest (and perhaps most famous) 11-year solar cycle variations down to periods of the order of minutes or even seconds (determined by current instrumental limits).

In recent years, images of the corona and parts of the heliosphere have become available in near-real time and at increasingly higher cadence. Recent investigations typically make use of these advances to study rapid-intensity fluctuations (of the order of minutes or even seconds). Our investigation instead focuses on much longer-term variations seen in coronal and heliospheric plasma. Periods of the order of an hour or longer have been recovered for several decades across a range of coronal instruments (*e.g.* by Ivanov, 1983; Foullon, Verwichte, and Nakariakov, 2004; Popescu *et al.*, 2005; Auchère *et al.*, 2014). The *Solar Dynamics Observatory* (SDO: Pesnell, Thompson, and Chamberlin, 2012) allows for continuous high-resolution and high-cadence measurements across a suite of instruments; this observatory has also contributed significantly to studies of long-period oscillatory features in recent years. Examples include Smirnova *et al.* (2013), who uncovered evidence of three- to seven-hour oscillatory periods using the *Heliospheric and Magnetic Imager* (HMI: Scherrer *et al.*, 2012) onboard SDO, while Froment *et al.* (2015) found four- to nine-hour periodic signatures using the *Atmospheric Imaging Assembly* (AIA: Lemen *et al.*, 2012).

Of particular interest for this investigation is the work of Telloni *et al.* (2013), who used wavelet analysis of light curves from the COR1 coronagraph (Howard *et al.*, 2008) onboard the STEREO observatory to reveal four- to eight-hour periodic variations in light intensity aligned with magnetic structures reaching out into the heliosphere. In this study, we look to extend the work of Telloni *et al.* (2013) by attempting to trace evidence of long-period intensity oscillations in the upper corona and heliosphere back to their apparent source(s) close to the solar surface.

As well as this scientific goal, we also examine how different noise models (and their associated confidence levels, above which oscillatory signals can be identified) fundamentally affect our findings, using the approach proposed by Auchère *et al.* (2016). In order to correctly distill true oscillatory behaviour from recovered Fourier or wavelet power spectra, one must first identify and remove noise contributions. White noise uniformly affects power spectra across all frequencies or periods, and it is accounted for by a uniform confidence level. A growing body of examples across a range of astrophysical phenomena, including X-ray binaries (van der Klis, 1995), Seyfert galaxies (Lawrence *et al.*, 1987; Markowitz *et al.*, 2003), gamma-ray bursts (Ukwatta *et al.*, 2009; Cenko *et al.*, 2010), and quasi-periodic pulsations (QPPs) during solar flares (*e.g.* Inglis, Ireland, and Dominique, 2015) yield power-law-like spectra. Contributions by erratic, aperiodic changes in brightness lead to spectral power $P$ of the form $P\propto f^{-\alpha}$ for frequencies $f$. This is a characteristic of *red noise*, which is an intrinsic property of the observed source. Spectra can be further complicated when detrending light curves prior to Fourier or wavelet analysis. Several recent articles (*e.g.* Gruber *et al.*, 2011; Inglis, Ireland, and Dominique, 2015; Ireland, McAteer, and Inglis, 2015; Auchère *et al.*, 2016) have invalidated several reported QPP observations (*e.g.* from Inglis and Nakariakov, 2009; Nakariakov *et al.*, 2010) that originally incorporated detrending and did not account for the power-law nature of the recovered spectra. Several methods have been proposed, *e.g.* by Vaughan (2005), Gruber *et al.* (2011), Auchère *et al.* (2016), Pugh, Broomhall, and Nakariakov (2017), in order to identify periodic signals in red-noise-dominated spectra. These methods typically comprise two distinct components: first the mean power as a function of frequency has to be estimated (based on fitting a power law, plus additional components). Various statistical tests may then be performed based on this mean power estimate, which determine what (if any) spectral features are statistically significant. In our work, we examine both the fitting of an estimated background power and subsequent statistical tests in our application to specific observational data.

Following a description of the data preparation and target region selection in Section 2, we detail our initial findings in Section 3.1. A reanalysis of the AIA data using a red-noise based model is then presented in Section 3.2, followed by the analysis of the STEREO data in Sections 3.3 to 3.5. The corresponding analysis of both the AIA and STEREO data using a white-noise-based model is presented for comparison in the Appendix. Discussion and conclusions are presented in Sections 4 and 5, respectively.

## Event Identification and Data Preparation

To study the propagation of long-period oscillatory signals out from the Sun into the inner heliosphere, we targeted an active region in close proximity to a coronal hole using a combination of EUV images and coronagraph measurements at the same time (and cadence). This would allow for visual identification of the initial structures close to the solar surface and a search for any related signals higher in the solar atmosphere.

**Figure 1:**
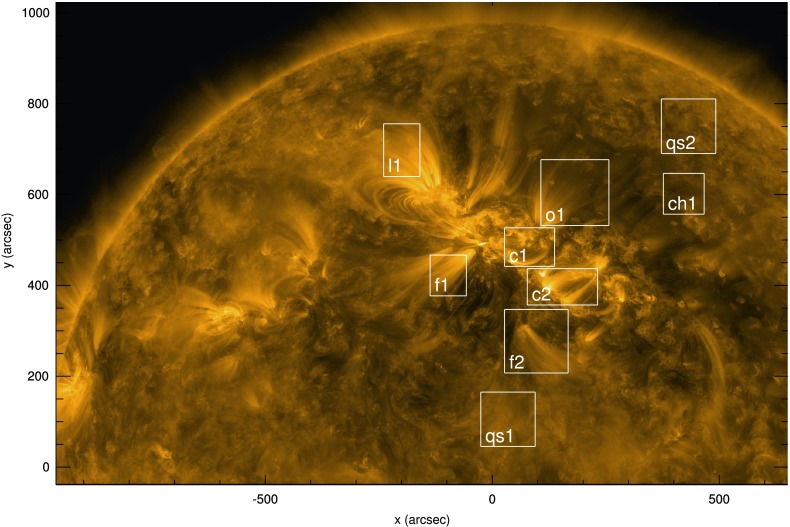
Identification of specific regions of interest on disk from the viewpoint of the $171~\mathring{\mathrm{A}}$ passband of SDO/AIA at 16:30:13 UT on 3 March 2011. Regions have been identified at the limb, then tracked in every frame until they reached disk centre. *White boxes* indicate features included in our study, labelled according to contents: the cores of both active regions (labelled c1 and c2) are surrounded boxes that sample fan loops (f1 and f2), closed loops (l1), and open structures (o1), together with a neighbouring equatorial coronal hole (ch1) and quiet-Sun (qs1 and qs2) regions.

The candidate region used in this study entered the field of view (FOV) of SDO/AIA on 25 February 2011 over the eastern solar limb. Images from AIA and later images from the *Extreme Ultraviolet Imager* (STEREO/EUVI: Wuelser *et al.*, 2004) show that both open and closed magnetic structures are present, and the target region is preceded by a large coronal hole structure, which extends almost to the solar equator. The area in question contains two active regions, catalogued as (from North to South) NOAA-11163 and NOAA-11164. STEREO-A was almost perpendicular to the Sun–Earth line at this time (at a separation angle of $87.4^{\circ}$) and hence the target region appears over the eastern limb of the Sun in EUVI-A images much later, on 2 March 2011. The dates noted here are approximate; the presence of open magnetic loop structures high into the solar atmosphere implies that some parts of this active region will be visible above the limb for some time before the active region core itself crosses onto the disk. An image of the target region near disk centre from AIA $171~\mathring{\mathrm{A}}$ can be seen in Figure 1.

We obtained level 1.5 data with a $15\text{-minute}$ cadence from the $171~\mathring{\mathrm{A}}$ and $193~\mathring{\mathrm{A}}$ AIA passbands, beginning on 27 February 2011 at 23:45:13 UT and ending on 3 March 2011 at 23:30:13 UT using the FOV seen in Figure 1. Each AIA dataset has been cleaned and co-aligned using the SolarSoft IDL AIA_prep command, before being de-rotated and using a cross-correlation technique to confirm spatial alignment of the 171 and $193~\mathring{\mathrm{A}}$ datasets. Finally, the time series for every pixel in both passbands have been interpolated to fill in any missing frames. The cadence was fixed at 15 minutes to suppress short (*e.g.* three- or five-minute) periods in the data.

Companion level 1.5 data were also obtained from several instruments of the *Sun Earth Connection Coronal and Heliospheric Investigation* (SECCHI: Howard *et al.*, 2008) instrument suite onboard STEREO-A, including EUVI and the white-light coronagraphs COR-1 and COR-2. For these instruments, we acquired data based on the times when the region in question would be at/close to the solar limb from the vantage point of the STEREO spacecraft. $195~\mathring{\mathrm{A}}$ EUVI, COR-1, and COR-2 data were all obtained at a 15-minute cadence over near-identical time windows running from 1 March to 3 March 2011. Specifically, EUVI data were recorded from 00:15:30 UT on March 1 until 23:45:30 UT on March 3. Similarly, COR1 and COR2 data run from 00:05:00 UT and 00:24:00 UT on 1 March until 23:50:00 UT and 23:39:00 UT on 3 March, respectively. Data from the remaining SECCHI instruments, the *Heliospheric Imagers* (HI-1 and HI-2), were also obtained from STEREO-A at this time, but were omitted from this investigation due to the restrictive image cadence (either 40 minutes in the case of HI-1 or 2 hours for HI-2). EUVI and COR datasets were prepared using the SolarSoft IDL SECCHI_prep command. Interpolation in time at each pixel was again used to fill in any missing frames and obtain a constant 15-minute cadence.

## Long-Period Intensity Oscillations

### Periodic Signal Analysis of SDO/AIA Data

Within the AIA FOV seen in Figure 1, we identified different categories of solar features and defined rectangular boxes that encapsulate them (in every frame), labelled according to contents. The boxes labelled c1 and c2 in Figure 1 contain loops at the core of the active regions, while f1 and f2 surround open fan-loop structures, qs1 and qs2 enclose regions of quiet Sun, ch1 is a region of coronal hole, o1 contains open loops associated with the northern active region, and l1 contains the edge of a set of closed loops that appear to gradually open (but whose connectivity remains unclear).

**Figure 2:**
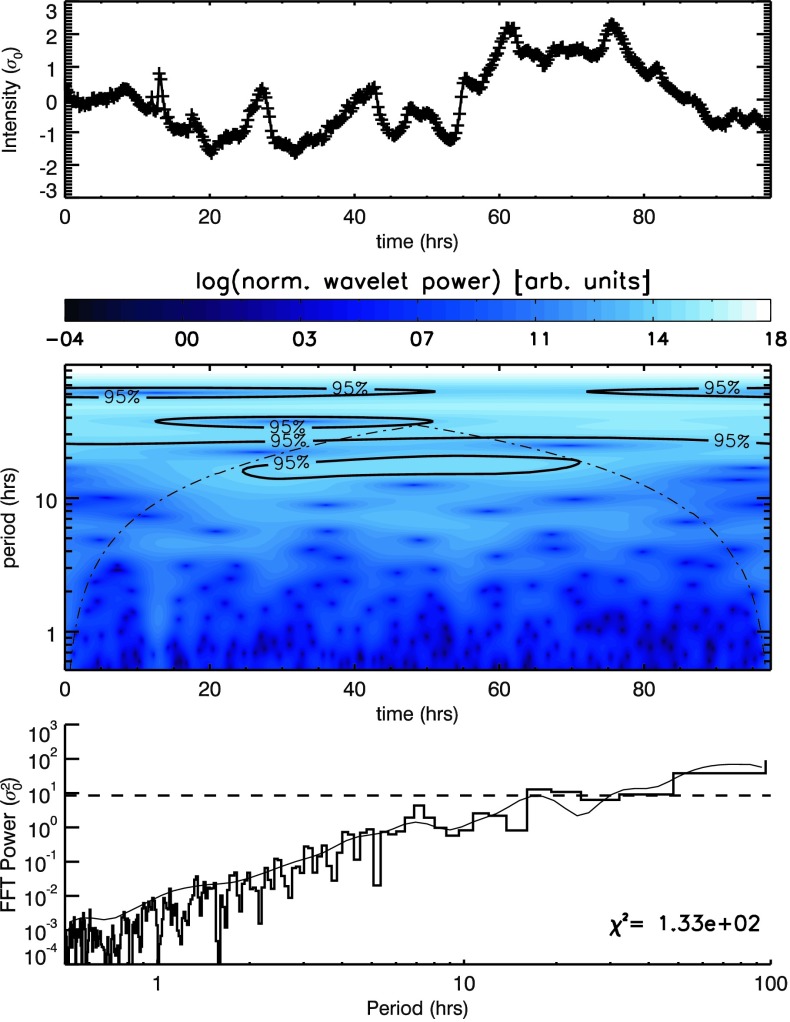
Signal (*top*) and resulting Morlet wavelet (*middle*) and Fourier power spectra (*bottom*) recovered by summing pixel intensities in box o1 in Figure 1. The *thin black line* in the bottom row indicates the time-averaged wavelet spectrum (*i.e.* the average of the middle row), while the *black histogram* indicates the FFT spectrum. Overlaid on the wavelet spectra are contours indicating the cone of influence (*dot-dashed line*), where edge effects begin to play a role, while the *labelled contours* (*middle*) and *horizontal dashed line* (*bottom*) indicate the corresponding $95\%$ white-noise confidence level.

We summed the intensity over all pixels in a single box in each frame, creating one light curve per box. An example of such a light curve can be seen in the top row of Figure 2 for the o1 box in the $171~\mathring{\mathrm{A}}$ passband (which has also been normalised to the variance of the total intensity from the average intensity). This signal was analysed to assess the likelihood that it contains periodic features using a (Morlet) wavelet analysis (middle row) or the standard IDL Fast Fourier Transform (FFT) routine (bottom row).

Both the wavelet and FFT analysis of the light curve from the o1 box contain peaks between 10 – 20 hours above the $95\%$ white-noise confidence level (and outside the cone-of-influence for the wavelet analysis). In addition, however, there are also significant changes in spectral power from short to long periods. This is particularly clear from the FFT analysis (bottom row of Figure 2), for which power increases by several orders of magnitude with period (appearing as power-law-like when shown using a logarithmic $x$- and $y$-axis). The same is true for the time-averaged wavelet spectra (seen as a thin black line closely following the FFT histogram in the bottom row of Figure 2).

Signatures of oscillatory behaviour are denoted by significant departures (in specific period bins) from the background variation in Fourier power. In white-noise-dominated spectra, this background is constant, and hence significant departures from this trend are easy to identify. In such cases, uniform confidence levels readily distinguish such large departures from any small, random variations in light intensity above this constant background. When the mean Fourier power itself varies significantly with period (as it does in the bottom row of Figure 2), uniform confidence levels are no longer able to clearly distinguish between any background variation in Fourier power and large departures from that background. For completeness (and for comparison with later results), we have also calculated the goodness-of-fit measure [$\chi^{2}$] when fitting the Fourier spectra (seen in the bottom row of Figure 2) with a uniform (white-noise) background power. The recovered value indicates an extremely poor fit. Thus the horizontal dashed line in the bottom row of Figure 2 (itself based on the assumption of white noise or a uniform mean Fourier power) is misleading. From it, one would conclude that several bins in the 10 – 100 hour range of periods contain Fourier power much greater than the background power. However, the power in these bins also fits the general background increase in Fourier power with period seen over the entire spectra. Such a trend in the background power is known as “red noise”. Similar variations in background power are present in every spectrum for each light curve (and in each passband) for every box identified in Figure 1, although with different slopes.

Because of the background variation seen in our spectra, we analysed each spectrum using confidence levels calculated above a background-noise model (which can accommodate red- and white-noise-dominated spectra as well as transients). For comparison, in the Appendix, we also illustrate the results we obtain using uniform (white-noise-only) confidence levels on detrended data. This allows us to apply and test the red-noise model with instruments other than AIA, and critically analyse the results of each approach, as well as compare our findings directly with those seen by, *e.g.*, Telloni *et al.* (2013).

### Periodic Signal Analysis of SDO/AIA Data Using the Red-Noise Model

The analysis and interpretation of Fourier and wavelet spectra in environments dominated by red noise have been studied for some years in a variety of contexts (*e.g.* Vaughan, 2005; Gruber *et al.*, 2011), but the technique has only recently started to gain traction in the analysis of light curves from solar environments/instruments. Several articles, particularly in the context of quasi-periodic pulsations (QPPs) associated with solar flares, now incorporate the fundamental power-law nature of power spectra in the analysis of coronal time series, but they use several different approaches (*e.g.* Ireland, McAteer, and Inglis, 2015; Inglis, Ireland, and Dominique, 2015; Auchère *et al.*, 2016; Pugh, Broomhall, and Nakariakov, 2017). We based our analysis on Auchère *et al.* (2016), who proposed a new method for deriving global confidence levels, and we tested it (together with an appropriate background-noise model) using long, high-cadence light curves derived from specific SDO/AIA observations. We used this method to reanalyse the results obtained in the previous section (as well as the STEREO results reported in the Appendix). To our knowledge, this is the first time this method has been used to semi-automatically analyse STEREO data.

**Figure 3:**
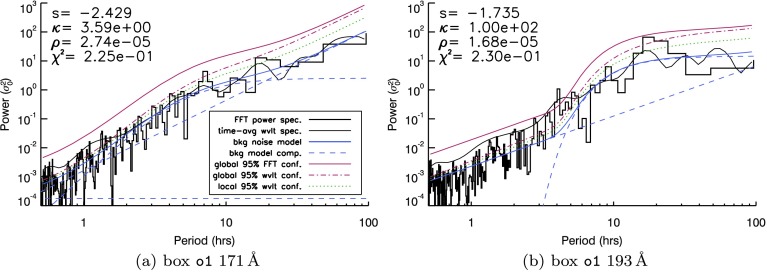
FFT spectra (*black histogram*) and time-averaged wavelet signal (*thin black line*) produced by light curves from the o1 box identified in Figure 1 at two different wavelengths. A brief guide to the remaining lines is given in the legend within (**a**). Expanding on this guide, each image contains a global $95\%$ red-noise confidence level (*red thick solid line*) that is based upon (and maintains the same distance above) the background-noise model fit to the Fourier spectrum (for the model given by Equation 1, with individual fit shown as a *thick blue line* and fit components shown as *blue dashed lines*). $95\%$ local (*green dotted line*) and global (*red dot-dashed line*) wavelet confidence levels are included for comparison. Values of components of Equation 1 for each fit are included with each spectrum (along with the resulting power law, $\kappa$-distribution, and white-noise fit components, seen as *blue dashed lines*), while the goodness-of-fit measure [${\chi^{2}}$] is also included in each example.

Returning to our target region $\mathtt{{o1}}$ in Figure 1, we again calculated the power spectral density for each signal in two AIA passbands in Figure 3. To calculate confidence levels, we now followed Auchère *et al.* (2016) in fitting a function $\sigma(\nu)$ to each power spectrum as a function of frequency $\nu$, as shown in Equation 1,

1 $$ \sigma(\nu)=A\nu^{s}+BK_{\rho}(\nu)+C. $$

Equation 1 contains contributions from a power-law dependency (with index $s$), humps in the data caused by transient, sporadic features (modelled using a kappa function $K_{\rho}(\nu)$,

$$K_{\rho}(\nu)= \biggl(1+\frac{\nu^{2}}{\kappa\rho^{2}} \biggr)^{-\frac{\kappa+1}{2}}, $$

with a width parameter $\rho$ and extent into the short-period range determined by $\kappa$), and a constant $C$ (which accounts for white-noise contributions). Equation 1 was then fitted to each individual power spectrum through the standard iterative least-squares fitting IDL routine MPFIT. Components of Equation 1 can play a lesser or greater role in each fit (or can be discarded entirely) through the values of $A$, $B$, and $C$ (and indeed $\kappa$, $\rho$, and $s$). For example, fitting a power law alone (*e.g.* Vaughan, 2005) could be achieved by a solution where $B=0$ and $C=0$, while a broken power law (*e.g.* Gruber *et al.*, 2011) could be reproduced by a solution where $B=0$ only.

**Figure 4:**
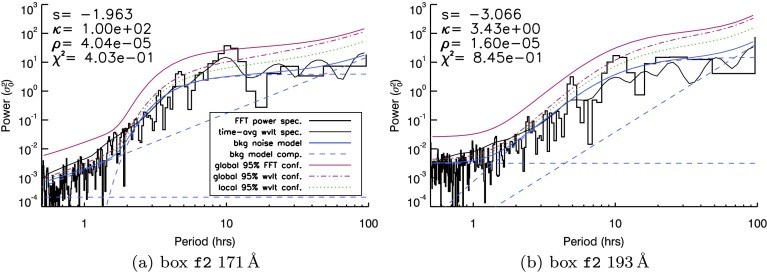
FFT spectra (*black histogram*) and time-averaged wavelet signal (*thin black line*) produced by light curves from the f2 box identified in Figure 1 in (**a**) $171~\mathring{\mathrm{A}}$ and (**b**) $193~\mathring{\mathrm{A}}$. A brief guide to the quantities in each image can be found in the legend in (**a**). A more detailed description of these quantities can be found in the caption of Figure 3.

**Figure 5:**
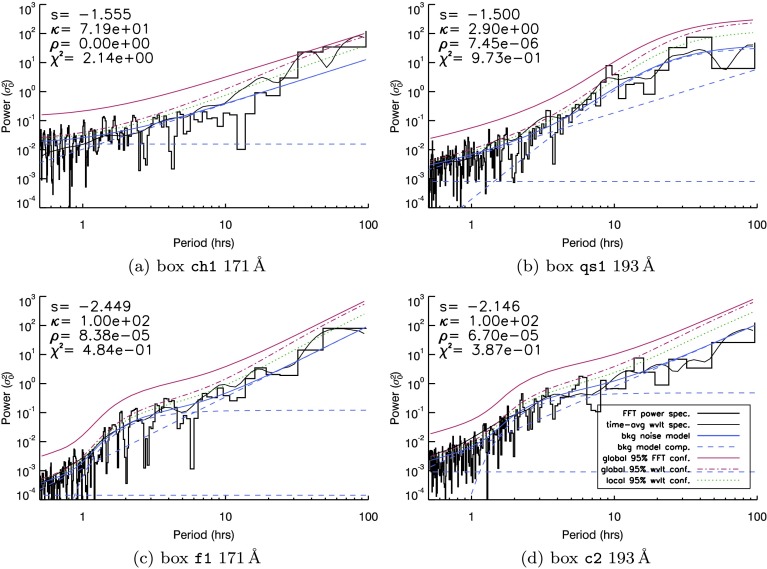
FFT spectra (*black histogram*) and time-averaged wavelet spectra (*thin black line*) produced by light curves from four boxes identified in Figure 1: (**a**) shows the ch1 box seen in $171~\mathring{\mathrm{A}}$, (**b**) the $193~\mathring{\mathrm{A}}$ qs1 box, (**c**) the $171~\mathring{\mathrm{A}}$ f1 box, and (**d**) the $193~\mathring{\mathrm{A}}$ c2 box. A brief guide to the quantities in each image can be found in the legend in (**b**). A more expansive description of these quantities accompanies the images in Figure 3.

Global confidence levels to the resulting fit were then generated using the method described by Auchère *et al.* (2016). Building upon earlier work (of, *e.g.*, Scargle, 1982; Gabriel *et al.*, 2002), this method proposes Monte Carlo-derived parameters to use as input into the IDL wavelet routine (of Torrence and Compo, 1998) in order to fully account for the number of degrees of freedom in the wavelet spectra. We first applied this technique to calculate confidence levels for the o1 box in Figure 3 before analysing light curves from the other AIA boxes (in Figures 4 and 5).

In Figure 3, the power spectral density as a function of period is shown (as a black histogram) for both the $171~\mathring{\mathrm{A}}$ (Figure 3a) and $193~\mathring{\mathrm{A}}$ (Figure 3b) AIA passbands, together with the time-averaged wavelet spectra (thin black line, as before). The mean power model fit of Equation 1 to the Fourier spectrum is shown in blue (with the power law, kappa distribution, and constant components of each fit seen as light blue dashed lines). Global $95\%$ confidence levels based on this mean power model (calculated using the method of Auchère *et al.*, 2016) are shown as a solid red line. For comparison, we also include the local wavelet $95\%$ confidence level derived by the Torrence and Compo (1998) wavelet code (but adapted to use the custom noise model of Equation 1) and the global wavelet $95\%$ confidence level derived by Auchère *et al.* (2016). However, Auchère *et al.* (2016) showed that the Torrence and Compo (1998) confidence levels can be underestimates, and hence lead to false-positive periodicity identifications.

Using Figure 3 as a typical example, both the local and global wavelet $95\%$ confidence levels converge towards the mean power estimate of the FFT at shorter periods in both cases. While the global wavelet confidence level consistently lies above the corresponding local confidence level, many spectral bins and parts of the time-averaged wavelet spectra often lie above both wavelet-based confidence levels, particularly at short periods. This could be attributed to mismatches in intensity at the beginning and end of the light curves influencing the (zero-padded) wavelet spectrum (see, *e.g.*, Auchère *et al.*, 2016). The global $95\%$ Fourier confidence levels derived from the mean power estimate using the noise model given in Equation 1 (in *e.g.* Figure 3) maintain a consistent gap above the fitted function across all periods. While we include both global Fourier and local/global wavelet confidence levels in any further examples, the tendency of the wavelet confidence levels to lie below the wavelet and Fourier spectra at short periods means that we used the global $95\%$ confidence level derived using the estimated power model as the most severe threshold above which periodic signals may be identified.

It is clear that the global confidence levels in Figure 3a do indeed impose a more severe test on the power spectra. Although several periods can be identified above the wavelet-derived confidence levels, no evidence of periodic behaviour appears above the global Fourier confidence levels for the fitted background-noise model in Figure 3a. The $\kappa$-distribution contributes broadly over the fitted model (blue) and dominates the confidence level (red) over periods from 2 – 20 hours. Meanwhile, in Figure 3b, the $\kappa\text{-distribution}$ component fit is taller and broader, dominating the background-noise model fit over periods from 5 – 100 hours. This change leaves a single FFT bin visible above confidence in the $193~\mathring{\mathrm{A}}$ spectrum, at a period of approximately four hours. The power-law index [$s$] obtained from the automatic fitting process, also differs in the two wavelengths: the $171~\mathring{\mathrm{A}}$ signal yields $s\approx-2.4$, while the $193~\mathring{\mathrm{A}}$ signal is shallower at $s\approx-1.7$. The fit of the noise model to the spectrum in the $193~\mathring{\mathrm{A}}$ case relies more heavily on the $\kappa$-component of Equation 1 than the $171~\mathring{\mathrm{A}}$ spectrum. Figure 3a is a closer fit to a simple power-law model (used by Gruber *et al.*, 2011), while Figure 3b appears closer to a broken power law plus additional humps, as per Equation 1.

The findings of the o1 box illustrate that the same underlying structures may yield relatively different background noise characteristics in different passbands. The same is true for box f2 (Figure 4), which contains our most likely positive detection of a periodic signature above confidence. In Figure 4a, two bins are recovered above confidence at periods close to ten hours in $171~\mathring{\mathrm{A}}$, at the centre of a Gaussian-like enhancement of several neighbouring bins above the mean power estimate. A similar spectral feature is visible at the ten-hour period mark in $193~\mathring{\mathrm{A}}$ in Figure 4b, but this crucially remains below the $95\%$ global confidence threshold. While the fit of the $\kappa$-component may be partially responsible for the bins seen above confidence in Figure 3b, the same cannot be said for the detection in Figure 4a, where the fitted $\kappa$-component provides a very good match to the background mean power.

We have analysed the remaining boxes seen in Figure 1, and we present a small sample of our findings in Figure 5 for four boxes in either $171~\mathring{\mathrm{A}}$ or $193~\mathring{\mathrm{A}}$, illustrating behaviour found in a coronal hole region (Figure 5a), a quiet-Sun region (Figure 5b), fan loops (Figure 5c), and active-region-core loops (Figure 5d).

In summary, there is scant evidence of any other periodic features visible above global confidence levels, although in all cases several peaks can be identified above the wavelet confidence levels. A single bin is just visible above the red line in Figures 5a and 5b. In the former case, the least-squares fitting-process does not include a $\kappa$-component in the fit to any part of the spectra. In the latter case, the $\kappa$-component has been matched to periods between 10 – 100 hours, which may overestimate the mean power in this region. Hence, the 8 – 9 hour peak in the qs1 box may indicate a significant detection. It is also worth noting that the qs1 box is located close to the f2 box, in which oscillatory signatures are identified at very similar periods (in Figure 4b). Both ch1 and qs1 boxes yield spectra with relatively large white-noise components. For Figure 5a, the white-noise component of the spectra lasts well into the 10 – 20-hour period range. The more “active” boxes (*e.g.* f1 and c2) yield spectra that are more broadly power-law-like, seen in Figures 5c and 5d. The $\kappa$-component for both of these fits covers periods between one to ten hours in both cases, while there is little/no visible white-noise component in these spectra.

Finally, as a part of the fitting process, we also obtained a measure of the goodness-of-fit of our background-noise model to each individual FFT spectrum. Labelled $\chi^{2}$, the automated procedure seeks to minimise this value in order to derive optimal parameters for Equation 1. Each image in Figures 3 – 5 is accompanied by a value of $\chi^{2}$. These values indicate that Figures 5c and 5d are the best fits to the spectra, while Figure 5a is the worst fit. Going forward, we use the value of $\chi^{2}$ as a guide, and we disregard fits whose $\chi^{2}$-values have become higher than a threshold (which we set to two), as a way to systematically rule out information gained using poorly fitting background-noise models. If applied to these spectra, this threshold would eliminate the $\mathtt{{ch1}}$ box results in Figure 5a due to the quality of fit.

### STEREO-A/EUVI

**Figure 6:**
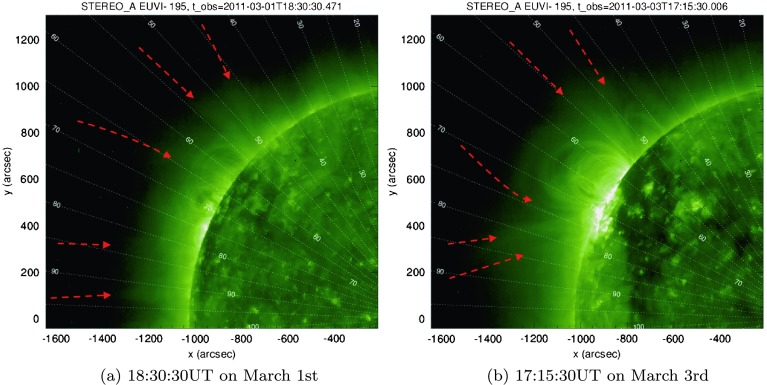
Reference STEREO-A/EUVI-$195~\mathring{\mathrm{A}}$ images taken close to the start and end of the time window of interest, observing the target active region transit over the solar limb. Highlighted in each image (using *red dashed arrows*) are likely open magnetic structures that may link to the outer heliosphere. Heliocentric lines of latitude are also overlaid as a guide.

We now turn our attention to periodic signals above the solar limb, first using ultra-violet images from STEREO-A/EUVI. Telloni *et al.* (2013) found that periodic signatures were associated with magnetic structures. In this investigation, our interest lies in open magnetic structures that extend out into the COR1 and COR2 fields-of-view and beyond. Using EUVI images, we note that several of the original “regions of interest” studied in Section 3.2 may be associated with such bands of open magnetic structures in Figure 6. These structures are indicated by red-dashed arrows and can be broadly grouped into three categories depending on their connectivity. The first category contains features originating below the southern active region with links to the equatorial plane (close to the line of $90^{\circ}$ latitude). The second category comprises open fan-loop structures emanating from above the northern active region (often close to the line of $50^{\circ}$ latitude). The third category appears between the latitudes of the previous categories, with apparent connections between/behind the two active regions, at/close to $65\,\mbox{--}\,75^{\circ}$ latitude. No additional active regions are present for several days after these dates at similar latitudes (see, *e.g.*, Figure 1).

The appearance of three bands of open structures is also to be expected from the configuration seen in Figure 1. It is likely that the equatorial open structures are related to box f2 (and possibly qs1), the northern structures are related to the region where box o1 (and perhaps l1) is located, while the features found between/behind the active regions match the latitude of box f1 well. A more definitive link between studied features and the local magnetic environment could be found using global magnetic field extrapolation models of the solar atmosphere in any future studies.

### STEREO-A/COR1

**Figure 7:**
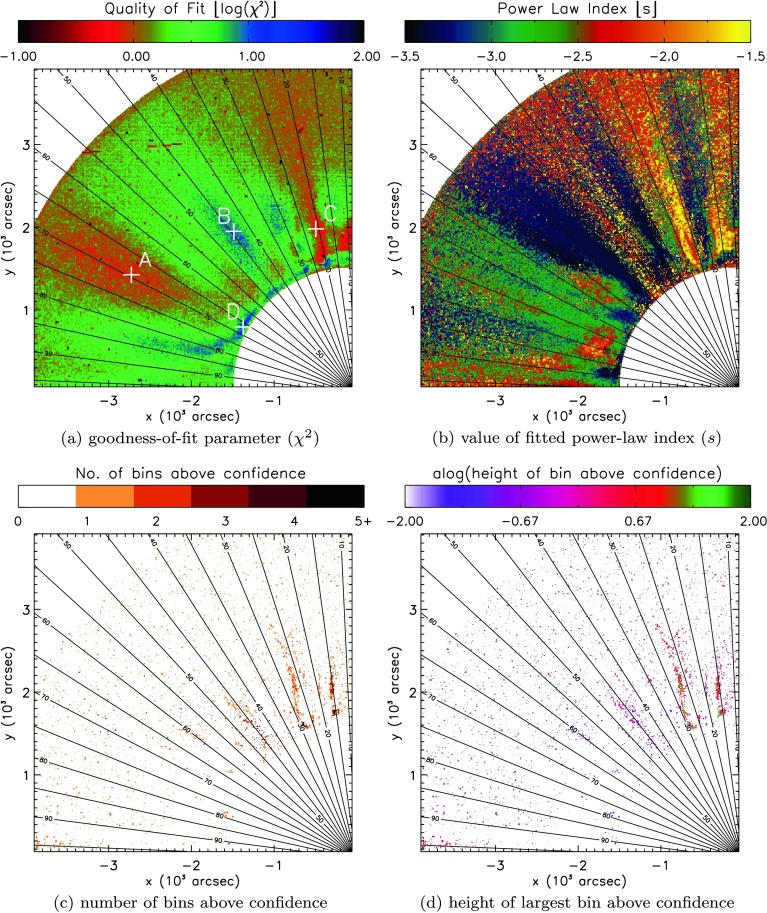
Maps illustrating (**a**) the goodness-of-fit $[\chi^{2}]$ and (**b**) the power-law index $[s]$, (**c**) the number of bins above confidence, and (**d**) the height of the largest bin above confidence recovered by least-squares fitting of Equation 1 to the FFT power spectra recovered from individual pixels in the STEREO/COR1 FOV. Example spectra found at four locations labelled A – D in (**a**) can be seen individually in Figure 9.

We now examine the light curve from every individual pixel in the COR1 FOV, subtracting a single average intensity prior to performing the Fourier and wavelet analysis. In this section, we again use the method proposed by Auchère *et al.* (2016). Results from an analysis using white-noise (uniform) confidence levels are presented in the Appendix for comparison. In order to establish the global, red-noise confidence levels, we repeated the process discussed in Section 3.2, but now automated for each pixel across the entire COR1 FOV. The sheer volume of data means that manual fitting/correction of the noise model to the spectra at each pixel becomes impossible. Instead, we automated the fitting procedure and inferred regional properties using maps of the FOV, where each pixel was assigned a colour according to a certain parameter value. The first set of maps is shown in Figure 7 for the COR1 instrument.

To critically assess the automated fitting of the confidence levels, we record the goodness-of-fit parameter $[\chi^{2}]$ (in Figure 7a) and (for a general sense of the fits) the power-law index $[s]$ (in Figure 7b). At the same time, our search for potential signatures of periodic behaviour relies upon counting the number of spectral bins above confidence (shown in Figure 7c) and recording the height above confidence of the largest bin (shown in Figure 7d) – both could be used to identify possible periodic signatures.

Several general trends are apparent from Figures 7a and 7b. The background-noise model given in Equation 1 produces a good fit to the data where $\chi^{2}$ is smallest. Three regions in Figure 7a produce the lowest values of $\chi^{2}$, between $65\,\mbox{--}\,75^{\circ}$ and $20\,\mbox{--}\,25^{\circ}$ latitude, and a small region close to the occulter at $10^{\circ}$. The first of these is reasonably well matched to one of the regions of open magnetic structures identified using EUVI in Section 3.3, while the final two regions are likely linked to open polar coronal hole regions. Our automated process failed to yield a good match to the spectra near the occulter at most other latitudes, while higher in the atmosphere, the match is poor in three specific regions, at approximately $30\,\mbox{--}\,35^{\circ}$, $45^{\circ}$, and $80\,\mbox{--}\,85^{\circ}$ (in Figure 7a). The final two of these regions are in close proximity to the remaining two regions of open magnetic structures seen in EUVI.

The fits to the spectra yield a broad range of power-law indices across the entire COR1 FOV, as evidenced by Figure 7b. Regions of similar $s$-values appear to be grouped largely by latitude (except at low heights at active region latitudes). The value of $s$ also tends to slowly decrease with radius at any given latitude. This decrease is likely to be an artefact of decreasing signal-to-noise ratio with radius, leaving the power spectra to flatten as white noise begins to dominate many frequencies.

To establish whether any possible periodic signatures persist from the low corona to the outer heliosphere, we incorporate spatial information through maps of the COR1 FOV that identify the number and height of any bins above fitted global confidence levels in Figures 7c and 7d. We cannot hope to study every spectrum at every pixel in COR1; instead, our objective here is to identify coherent groups of pixels aligned with specific structures along which periodic signals may be present, before investigating these in more detail and comparing them with the SDO/AIA results.

Both Figure 7c and Figure 7d highlight several groupings of frequency bins that lie above the fitted (global) confidence levels. Approximately three groups can be identified close to the polar region, with a further possible cluster of pixels close to $95^{\circ}$ at the edge of the FOV. The polar groupings appear at approximately $15^{\circ}$, $25\,\mbox{--}\,30^{\circ}$, and $45^{\circ}$ latitude. Of the polar groups, the $45^{\circ}$ group appears much more sporadic in nature, and it only barely reaches above the fitted confidence levels. Again we point out that the global confidence levels impose a very strict test, where many more periods in the power spectra lie above the corresponding local confidence levels.

**Figure 8:**
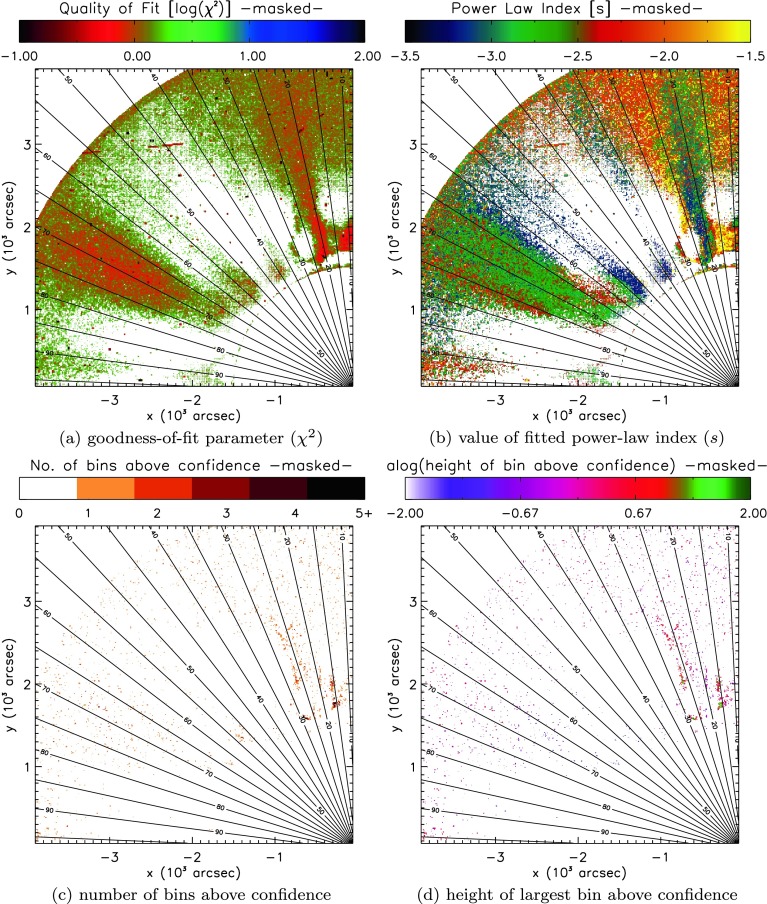
Masked version of Figure 7, only showing quantities where $\chi^{2}<2$, *i.e.* where the fitted function is found to most closely resemble the background spectra. Individual maps illustrate (**a**) the goodness-of-fit $[\chi^{2}]$, (**b**) the power-law index $[s]$, (**c**) the number of bins above confidence, and (**d**) the height of the largest bin above confidence recovered by least-squares fitting of Equation 1 to the FFT power spectra recovered from individual pixels in the STEREO/COR1 FOV.

Focusing on the remaining two polar “bands” of possible periodic features, we must first clarify if these bands lie in regions where Equation 1 is a good fit to the data. In order to do so, in Figure 8 we mask the findings shown in Figure 7 to only show regions where the goodness-of-fit measure $[\chi^{2}]$ is less than two (or where $\log(\chi^{2})<0.30$), suggesting a good fit of the background-noise model to the recovered spectra.

Figure 8 reveals a rather different picture of the COR1 spectra. Once again, we identify three regions where Equation 1 provides a good fit to the recovered spectra. Each appears to have subtly different properties in the power-law index, seen in Figure 8b. The region at $70^{\circ}$ commonly yields indices close to −2.5, while the region at $20^{\circ}$ typically contains power indices across a broader range ($-3\lesssim s\lesssim-2$), with the small polar region ($10^{\circ}$) yielding flatter index values ($-2\lesssim s\lesssim-1.5$).

However, our search for coherent features supporting possible periodic features undergoes a dramatic change when masking the number and height of bins to show only values where $\chi^{2}<2$. Almost all of the bands of features identified in Figures 7c and 7d no longer remain present or as coherent in Figures 8c and 8d. The most likely region to identify spectral bins above confidence remains the polar region, but no clear picture emerges of a specific structure supporting periodic features.

**Figure 9:**
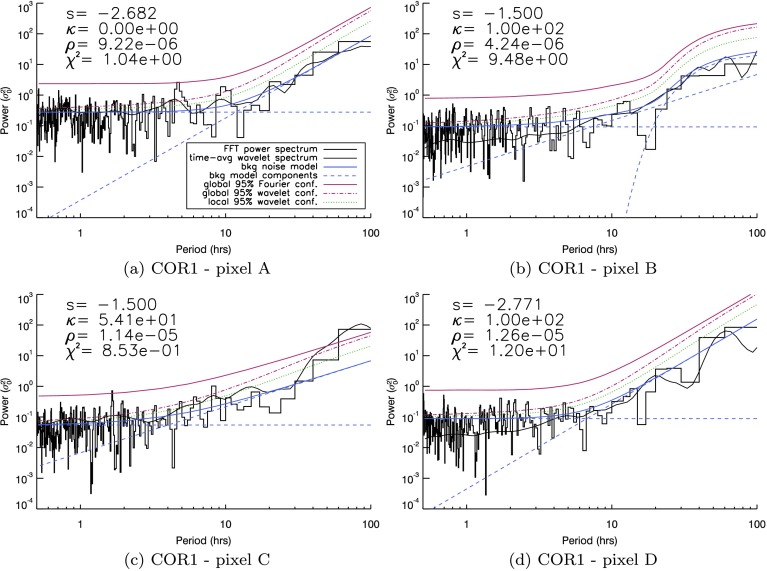
FFT spectra (*black histogram*) and time-averaged wavelet spectra (*thin black line*) for four individual pixels in the STEREO/COR1 FOV, labelled A – D in Figure 7a, with global red-noise $95\%$ confidence levels (*red solid line*) above background-noise model fit to Fourier spectrum (using model given by Equation 1, with overall fit seen in *blue* and fit components seen as *blue-dashed lines*). Local (*green dotted line*) and global (*red-dotted line*) wavelet $95\%$ confidence levels are included for comparison. Values of components of the fit are printed with each spectrum, along with ${\chi^{2}.}$

In Figure 9, we show examples of the spectral analysis in four individual pixels in the COR1 FOV (labelled A – D in Figure 7a). In general, we see that these particular power spectra appear “whiter” (or flatter) up to relatively long periods (of the order of perhaps 20 hours). For pixel A, there is a good fit for the noise model to the power spectrum and a single peak that is identifiable just above the associated global confidence level. For this pixel (as well as pixels C and D), the kappa function does not appear to be present in the fitted noise model. In contrast, for pixel B, the kappa function dominates the noise model for longer periods and the red-noise component is almost absent. Pixels B and D are classed as bad fits for the noise model (with ${\chi^{2}}\approx9.5$ and 12, respectively), possibly due to the presence of very deep bins in the power spectra. Other than perhaps being “whiter” than the SDO/AIA spectral examples, there does not appear to be a general trend in the behaviour of the power spectra in the COR1 FOV or in the fitting of the noise model.

### STEREO-A/COR2

**Figure 10:**
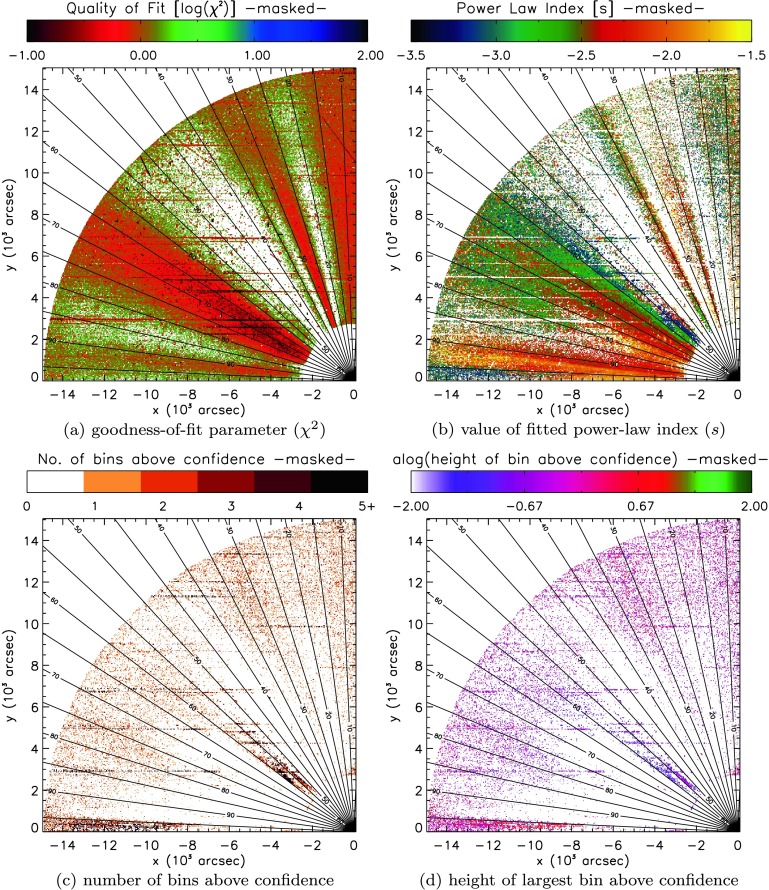
Maps illustrating fitting parameters in the COR2 FOV, only showing quantities where $\chi^{2}<2$, *i.e.* where the fitted function is found to most closely resemble the background spectra. Individual maps illustrate (**a**) the goodness-of-fit $[\chi^{2}]$, (**b**) the power-law index $[s]$, (**c**) the number of bins above confidence, and (**d**) the height of the largest bin above confidence recovered by least-squares fitting of Equation 1 to the FFT power spectra recovered from individual pixels in the STEREO/COR2 FOV.

Finally, to complete the picture of the corona and inner heliosphere, we apply the same technique to light curves from pixels from the COR2 FOV in Figure 10, where (as before) we display the goodness-of-fit measure $[\chi^{2}]$ of our fitted background-noise model (in Figure 10a), the power-law index (in Figure 10b), and the number of bins above the fitted global confidence level (Figure 10c) together with the height of the tallest bin above confidence (Figure 10d). For brevity, each of these has been masked to only show information where $\chi^{2}<2$ (or $\log(\chi^{2})<0.3$).

Using Figure 10, we first note that background starlight can interfere with the fitting process, leaving horizontal streaks in our maps. We also see similarities with the COR1 maps seen earlier. Our background-noise model (Equation 1) yields a good fit to the spectra in four bands in Figure 10a. Three of these bands are well matched to features seen in Figure 7a and Figure 8a, close to latitudes of $10^{\circ}$, $25\,\mbox{--}\,30^{\circ}$, and $60\,\mbox{--}\,80^{\circ}$. A fourth band, between $90\,\mbox{--}\,95^{\circ}$ latitude (near the Equator) was not visible in Figure 8a, but the incursion of a nearby (blue) region of poor fitting in Figure 7a may have obfuscated this feature close to the solar surface.

While there is no striking overall behaviour in each of these regions, for example in the value of the power-law index seen in Figure 10b, several minor trends are apparent. The region above the pole ($10^{\circ}$ latitude) contains some of the steepest *and* shallowest power-law indices recovered in by our fitting technique. The band at $25\,\mbox{--}\,30^{\circ}$ typically yields $-2.5< s<-1.5$, which is similar to the band seen at $90\,\mbox{--}\,95^{\circ}$. There also appears to be a latitudinal dependence of power-law index in each region, with values becoming progressively more negative moving polewards through each band. This is particularly notable in the broadest region (at $60\,\mbox{--}\,80^{\circ}$), but appears to be present in all bands in Figure 10b, except for the polar band (close to $10^{\circ}$).

Returning once more to our search for possible periodic signatures, Figures 10c and 10d now suggest that the band close to the Equator contains many pixels where power remains above confidence and that the difference between the height of the largest bin and the confidence level is greatest. This may be linked to the detection of a ten-hour periodic signature in the f2 box discussed in Section 3.2, but no similar feature is seen in COR1. Two other bands appear between $50\,\mbox{--}\,60^{\circ}$ that may contain many bins above confidence, but the difference between the power in the largest bin and the confidence levels in these features is much smaller than in the equatorial band. This strongly contrasts the view from COR1, where the only region containing coherent groups of pixels with bins above confidence was located close to the pole ($30\,\mbox{--}\,10^{\circ}$). Somewhat counter-intuitively, many more COR2 pixels containing bins above confidence are often visible closer to the outer edge of the FOV. This may be related to spectral whitening, as the signal-to-noise ratio decreases with height, and it is likely to be an artefact of the fitting of the noise levels. As these signals are not relevant to our study tracing periodic signatures magnetic features, we have not examined these pixels in detail.

## Discussion

### Recovery of Periodic Signatures

The aim of our investigation was to trace and analyse periodic signatures from the solar surface out into the heliosphere along visible open magnetic structures. Using wavelet analysis of light curves from COR1, Telloni *et al.* (2013) found periodic signatures in the four- to eight-hour period range to permeate the solar corona (at that time). These signatures often outlined spatially coherent fluctuating features aligned with the local magnetic field. Using the same instruments with co-temporal SDO/AIA images, we aimed to identify the origin of similar periodic fluctuations to specific features in the solar corona.

Our initial analysis of the AIA and STEREO data used the common assumption of a white-noise model, corresponding to uniform confidence levels for the power spectra. In addition, we originally detrended the data, again a widely used technique to remove slow variations or long-term trends from the data. This initial analysis revealed consistent periodic signatures in the AIA and STEREO data that aligned with magnetic structures (as in Telloni *et al.*, 2013). Crucially, however, this initial analysis of the power spectral density of both large regions in AIA and individual COR pixels also indicated that our spectra contain large variations in mean Fourier power. A growing recent consensus (*e.g.* Inglis, Ireland, and Dominique, 2015; Ireland, McAteer, and Inglis, 2015; Auchère *et al.*, 2016) strongly cautions against the application of white-noise confidence levels for similar time series that have also been detrended, in the context of solar oscillations.

Upon applying the red-noise background model to our spectra, three individual AIA boxes contained possible periodic detections above red-noise (global Fourier) confidence levels. Of these, the strongest evidence of a periodic signal occurs with a ten-hour period in the f2 box (seen in Figure 4a). In addition, recovery of four-hour and nine-hour periods in the o1 box (in Figure 3) and the qs1 box (in Figure 5b) lie at the edges of the typical range of periods reported in Telloni *et al.* (2013), but all are well within the range of periods previously recovered for a range of solar coronal structures (*e.g.* Ivanov, 1983; Foullon, Verwichte, and Nakariakov, 2004; Smirnova *et al.*, 2013; Auchère *et al.*, 2014). Furthermore, the o1 and qs1 detections are both marginally above global confidence levels and rely on the form of the fitted background power derived from Equation 1. The vast majority of AIA passbands and boxes that we have studied contain periodicities that lie above white-noise and/or wavelet-based local and global confidence levels but do not contain any periodicities above fitted global red-noise confidence levels.

This study represents the first attempt to apply the analysis proposed by Auchère *et al.* (2016) to observational data from STEREO. Moving from the SDO/AIA to the STEREO/COR fields of view, a similar picture emerges, of only sporadic and spatially uncorrelated pixels where periodic signatures are detected when using global confidence levels associated with a generic noise model combining white-noise, red-noise, and transients contributions fitted to the spectral power. These detections do not form spatially coherent features in the manner seen in Telloni *et al.* (2013), particularly at active region latitudes. The most promising coherent regions where bins are regularly found above confidence (*e.g.* $15\,\mbox{--}\,30^{\circ}$ latitudes in COR1, seen in Figures 7c and 7d) include large regions of poor fits to the data, and they are made much less clear by masking the images to only display pixels where $\chi^{2}<2$ (Figures 8c and 8d). Results from COR2 present yet another different picture, with three highly radial bands of periodic features visible in the FOV in Figures 10c and 10d. Of these, the band closest to the Equator contains more bins above confidence, and larger differences between the power in those bins and the height of the confidence level. The latitude of this band is also similar to that of the f2 box that is the likeliest candidate at the surface to support periodic features with periods close to ten hours. Two other bands, at $55\,\mbox{--}\,60^{\circ}$, contain power much closer to the confidence level. All three bands do not appear to be related to features in the COR1 FOV. Overall, there is scant evidence for coherent periodic behaviour in related (magnetic) regions/structures from our multi-instrumental viewpoint, using the global confidence levels generated by the mean power model proposed by Auchère *et al.* (2016).

**Figure 11:**
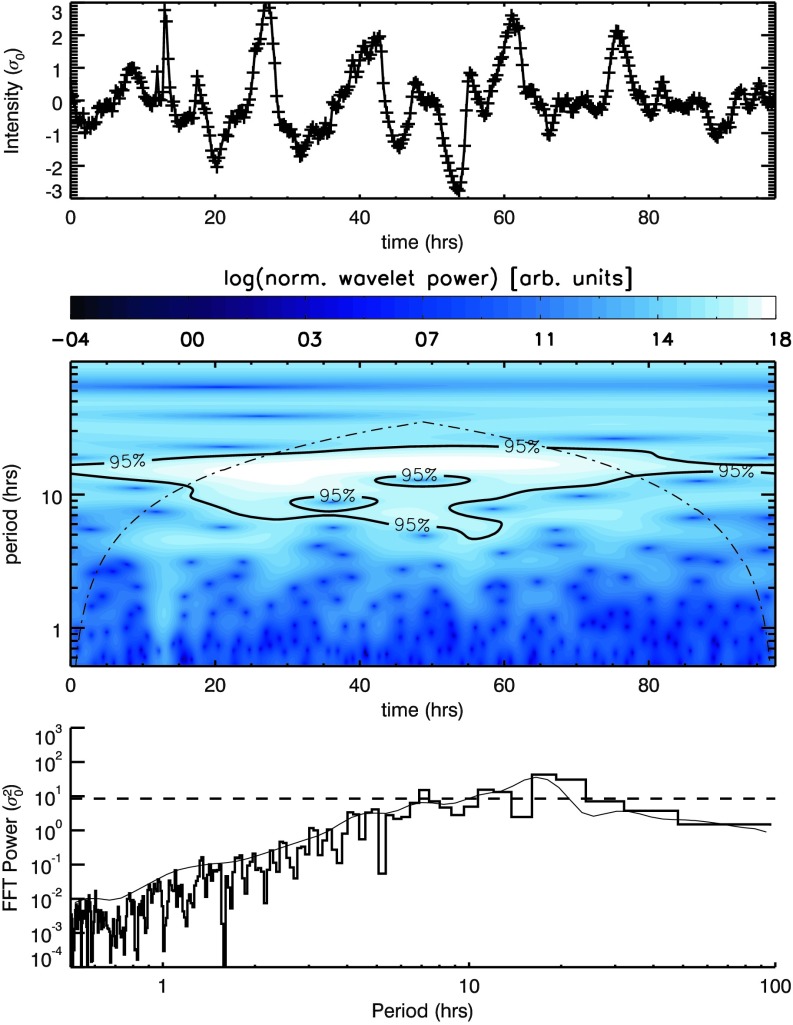
Signals (*top*) and resulting Morlet wavelet (*middle*) and Fourier power spectra (*bottom*) recovered by summing pixel intensities in box o1 in Figure 1, using the total intensity with a 15-hour smoothed signal subtracted from it. Overlaid on the wavelet spectra are contours indicating the location of the $95\%$ confidence threshold and the cone of influence (*dot-dashed line*) indicating the range of influence of edge effects. The *thin black line* included with the power spectra indicates the time-averaged wavelet spectrum, while the *horizontal dashed line* indicates the corresponding $95\%$ confidence level.

**Figure 12:**
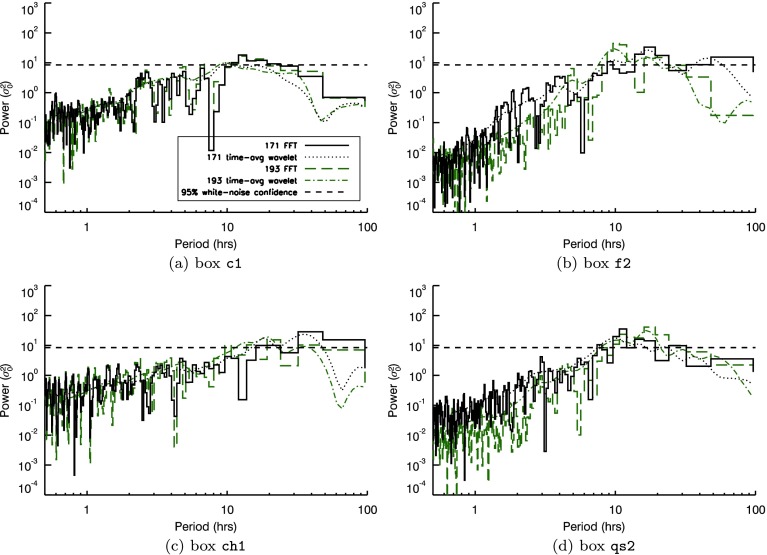
Power spectral density recovered from smooth-subtracted time series found by summing pixel intensities in (**a**) box c1, (**b**) box f2, (**c**) box ch1, (**d**) box qs2 (shown in Figure 1) for both 171 (*black solid histogram*) and $193~\mathring{\mathrm{A}}$ (*green dashed histogram*) passbands. The *smooth dotted* or *dot-dashed lines* indicate the time-averaged wavelet spectra for the $171~\mathring{\mathrm{A}}$ or $193~\mathring{\mathrm{A}}$ passbands, respectively, while the *black horizontal dashed line* indicates the $95\%$ white-noise confidence threshold.

Telloni *et al.* (2013) applied white-noise confidence levels to wavelet spectra from COR1 light curves that have been filtered to suppress periods below 2.5 hours and above 10 hours. This is known as detrending and is a commonly used technique to flatten/whiten spectra over specific period ranges (often for periods outside the range of interest or where edge effects, identified by the cone-of-influence of wavelet spectra, may significantly contribute to recovered power). These authors also stated that “In many astrophysical applications, it is a good choice to adopt as a background spectrum a flat spectrum”. In the Appendix, we highlight several alternative findings when we repeat our investigation using uniform (white noise) confidence levels applied to spectra that have also been detrended (in our case for periods at/above 15 hours). Using this different method, we regularly recover spectral peaks above uniform confidence levels at periods from 6 – 15 hours in Figures 11 and 12. Each AIA box identified in Figure 1 contains such peaks, while in both STEREO instruments, the ratio of period power in the 6 – 9 and 9 – 13-hour period ranges in each pixel closely matches latitudes identified in EUVI (in Figure 6) and white-light images obtained from the C2 and C3 instruments as part of the *Large Angle and Spectrometric Coronagraph* (LASCO: Brueckner *et al.*, 1995) onboard the *Solar and Heliospheric Observatory* (SOHO: Domingo, Fleck, and Poland, 1995) satellite, where open magnetic structures can be seen/inferred.

**Figure 13:**
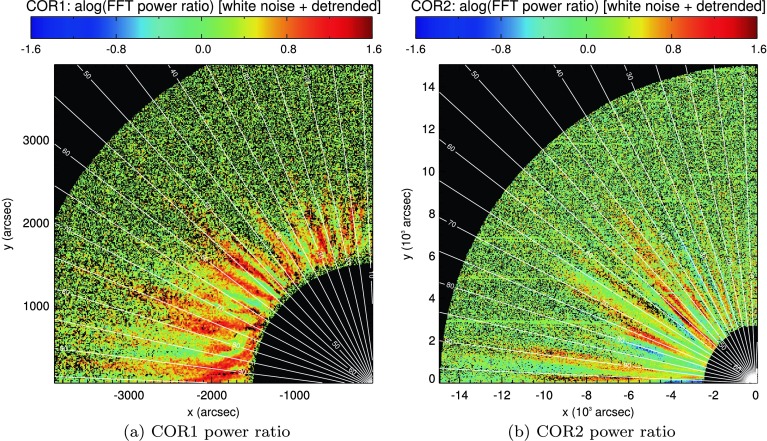
Ratio of peak Fourier power in the 9 – 13-hour to 6 – 9-hour period ranges, as seen by COR1 and COR2 onboard STEREO-A, based on smooth-subtracted time series of individual pixels in each instrument. *Black pixels* indicate locations where the FFT peak in either period bin was below the $95\%$ confidence interval.

The use of detrending in solar red-noise-dominated spectra has recently been scrutinised (*e.g.* Inglis, Ireland, and Dominique, 2015; Ireland, McAteer, and Inglis, 2015; Auchère *et al.*, 2016), noting the likelihood of false detections of periodic features close to the cutoff frequency of the detrending filter. In the Appendix, we note that some of the bins observed above confidence lie close to but below the 15-hour period of the detrending filter. This is, in some sense, to be expected. At the start of our investigation, we noted that the spectral power in both instruments varies considerably with period, and with power removed at/above 15-hour periods, our spectra will likely peak close to this value. It is also noteworthy that the upper detrending cutoff period in Telloni *et al.* (2013) lies at 10 hours, while detections are identified between 4 – 8 hours. Like our results in the Appendix, this was achieved through white-noise confidence levels applied to detrended spectra. However, such periodic signal detections cannot be simply dismissed *a priori*. These signatures also spread some distance from the cutoff frequency (ranging from 6 hours to 20 – 30 hours in some of the cases presented in the Appendix). The exact range of influence of our chosen detrending filter is unclear. While the removal of Fourier power at 15 hours or longer would amplify periods below but close to 15 hours, it is not obvious if/how this would extend to periods of 6 hours or below. Detections above the cutoff must maintain considerable Fourier power to remain after detrending. The STEREO data seen in Figure 13 show several coherent regions where shorter-period FFT power is much greater than longer-period power, in direct contrast to the red-noise picture. The period ranges and bin sizes used in this part of the study are relatively small and close together, and they could be similarly interpreted as local structure in spectral power. Nevertheless, not all periodic signatures found using white-noise confidence levels above detrended data can be easily dismissed.

### Red-Noise Background Model Fitting

The global confidence levels based upon the mean-power model used here impose a severe threshold on potentially significant periodicities in the power spectra. Our choices of red-noise background model and fitting technique are also inherently linked to our findings in Sections 3.2 to 3.5. In this investigation, we fit a specific background-noise model (Equation 1) to our spectra. This model incorporates features seen in other investigations (a power-law component, contributions from white noise, and sporadic transient humps) and hence is considered to be relatively generic. Indeed, earlier investigations into red-noise fitted confidence levels above a power law or broken power law (*e.g.* Vaughan, 2005; Gruber *et al.*, 2011). Our recovered power-law slope values are consistent with previous studies and might be associated with turbulence (*e.g.* Ireland, McAteer, and Inglis, 2015) or an impulsively heated corona (*e.g.* Aschwanden *et al.*, 2016, and references therein). Other effects, including line-of-sight superposition, also affect our recovered power-index values.

More recently, Auchère *et al.* (2016) included the kappa component seen in Equation 1, reasoning that this inclusion provides a better match to specific spectra over a certain range of frequencies. We have shown (using, *e.g.*, Figure 5) that while this is true in some cases, this “hump” is not present in many other examples, and the fitting process can mistake large parts of the spectra for this component, which was designed to present only as a small inclusion over a narrow range of frequencies. This component was originally introduced to account for macroscopic transient events (eruptions, for example). While no coronal-mass ejection-producing events are associated with our region of interest, the northern AR is associated with several small GOES flare detections (the largest of which was classed M1.1, and the majority of which were C-class events) during our observation window. How these events would affect each time series on a pixel-by-pixel basis in different instruments is unclear. However, it is vital to be able to distinguish between spectral contributions made by variations in mean power, noise, and true oscillatory signatures. A “hump” is clearly not present in every time series. If present, can the fitted spectral “hump” be better constrained *prior* to automated fitting? Furthermore, the physical justification of aspects of this model are unclear. What causes the variations in power-law index in neighbouring regions (and even pixels)? If a “hump” is identified in the power model, can its appearance be attributed to a specific (transient) feature? Transient features have been shown to trigger kink oscillations in coronal loops: would these be identifiable above confidence or masked by the inclusion of the hump in background power? Our analysis suggests that deriving alternative noise models and parameter regimes (particularly those that lend themselves to automation) may be necessary when studying different target regions or using different instruments.

Several additional factors influence our red-noise results. The numerical procedure refines a set of initial parameter “guesses” depending on the resulting fit of Equation 1 to the spectra before deciding upon the optimal parameter regime, determined by the goodness-of-fit $[\chi^{2}]$. We repeated this process for a range of different initial parameters per box, retaining only the set that recovered the fit with the lowest value of $\chi^{2}$ in each case. For the AIA investigation, the initial guesses were found by examining the fit of Equation 1 to each of the spectra by eye. All fits were highly dependent on these initial parameter values. Furthermore, the background-noise model fits benefit from extended spectral range (achieved by extending the dataset length or decreasing the cadence). However, upcoming missions (*e.g.* *Solar Orbiter* and the *Parker Solar Probe*) may not have this option.

Second, it is unclear whether the background-noise model and initial parameters chosen for our automated analysis are appropriate to derive accurate confidence thresholds for true periodic signatures. Masking our COR1 and COR2 fields of view by the goodness-of-fit $[\chi^{2}]$ reveals large regions where the analysis has failed to provide a good match to the background noise in each pixel. There is a clear need for automation in STEREO data; in AIA, it is relatively easy to broadly group features through a visual comparison, as we have done. Conversely, it is very difficult to visually identify specific features in COR (let alone identify and group related features). This automated method clearly defines regions of correlated behaviour with minimal effort. However, refinement is needed before it may reliably identify periodic signatures. Would a smoothed form of the spectra, or indeed some form of time-averaged wavelet spectra, be more reliable in cases where one cannot hope to check every instance of background-noise model fit to the spectra by eye?

## Conclusions

This study aimed to examine periodicities in intensity data using a multi-instrumental (co-temporal and co-spatial) survey of magnetic structures associated with a specific active region extending out from the corona into the inner heliosphere. Although several periods are readily identifiable in the power spectra using white-noise confidence levels (see the Appendix), the power spectra also show significant changes in spectral power from short to long periods, implying that confidence levels based on a red-noise model are needed (see also *e.g.* Ireland, McAteer, and Inglis, 2015; Inglis, Ireland, and Dominique, 2015; Auchère *et al.*, 2016).

The implementation of global red-noise confidence levels to solar data is non-trivial; we applied a recently proposed technique (Auchère *et al.*, 2016) to investigate the presence of periodic features in different general environments (active-region loops, open/fan loops, quiet Sun, coronal hole, *etc*.). Several environments showed possible evidence of periodic signatures in the four- to ten-hour period range, but more often, minimal evidence of periodic behaviour was recovered. General trends were also found as part of the background-noise model fitting, where more active coronal regions maintained steeper spectra with smaller contributions from white-noise components than more quiescent (*e.g.* quiet-Sun and coronal-hole) regions.

Extending our analysis to the COR1 and COR2 instruments onboard STEREO using the approach of Auchère *et al.* (2016) required fully automated fitting of the background-noise model to individual pixel spectra across the entire FOV. Using the resulting global confidence level fits to this model, there is scant evidence that specific magnetic structures in this dataset support spatially correlated periodic signatures into the heliosphere, particularly in COR1. This result is strikingly contrary to investigations that use white-noise confidence levels above detrended data (or local confidence levels), where evidence of long-period oscillations is relatively abundant, both close to the solar surface and above the limb.

Implementation of red-noise background model fitting and associated confidence level calculation still requires refinement, particularly in applications where automation is necessary. Many regions show a poor match of the automated model fit to the spectra, suggesting that either a different model or a better way to derive parameter fits may be necessary in future studies. In regions where good matches were recovered, the general characteristics identified in SDO/AIA red-noise-dominated spectra are also present in COR1 and COR2.

Various extensions to this work are readily apparent. This investigation is certainly worth repeating using observations of a region where strong oscillatory behaviour is known to be present in the corona (above red-noise confidence levels), which could then be studied from other points of view in the same manner as our approach here (*e.g.* by STEREO). Despite its seemingly generic nature, the choice of background-noise model here is lacking both in robustness and physical justification. A vital question is how closely the background-noise model should match the spectra. Would smoothed FFT spectra, time-averaged wavelet spectra, principal component analysis, or Thomson’s multitaper method (*e.g.* Viall *et al.*, 2010) provide a more robust, general background trend? The sheer quantity of data output from current and future instruments suggests that manual fitting of models to individual spectra is rapidly becoming insufficient. Automated fitting procedures (like this one) must be further refined in order to correctly identify periodic features in high-volume instruments, which may also be constrained by cadence and/or observing length.

## Appendix: Uniform Confidence Levels and Detrending

Several studies have recently reported periodicities in magnetic structures in the extended solar atmosphere (*e.g.* Viall *et al.*, 2010; Telloni *et al.*, 2013). White-noise confidence levels are a commonly used assumption in such studies (*e.g.* Telloni *et al.*, 2013). To allow a comparison with these particular studies and with the results obtained following the approach proposed by Auchère *et al.* (2016), we here repeat the AIA and STEREO data analysis using such uniform, white-noise confidence levels. We also detrend the original data.

Detrending is a commonly used technique to remove a slow variation (or long-term trend) from a time series. Any resulting Fourier analysis of detrended data is not significantly affected at periods below the detrending cutoff (but detrending can introduce small ripples in the spectra below this cutoff in some cases). Detrending deforms the power-law-like nature of red-noise-dominated spectra, requiring that any mean power model of the spectra must also take into account the shape of the detrending filter. For clarity, we did not use detrending when applying the method of Auchère *et al.* (2016) in the main body of our investigation, but we include it here to illustrate how its use can affect identification of periodic features above white-noise confidence levels.

### A.1 SDO/AIA Results

To detrend the AIA data, we subtracted a 15-hour smoothed version from the original light curve *prior* to the wavelet or FFT analysis. Figure 11 displays the wavelet and FFT results for the o1 box. In Figure 2, the dominant periodicities are almost all inside the cone of influence (COI – represented by the dot-dashed line in the wavelet results), indicating that edge effects (close to the start or end of the time series) influence the recovered peaks. The FFT spectrum of the detrended signal (Figure 11) now contains several peaks above the uniform confidence interval between 6 and 15 hours, which are also inside the COI for the wavelet analysis. The 15-hour smooth subtraction in this case removes the influence of signals with periods that begin to approach the length of the dataset and hence would fall inside the wavelet COI. Using different periods of smooth subtraction, we confirmed that this detrending of the original light curve does not affect period bins shorter than the length of smooth; longer periods are suppressed, while shorter periods remain unaffected. The FFT results for several of the other AIA boxes can be seen in Figure 12. Using this method for our AIA data analysis, we find several peaks in the range of 6 – 15 hours in both the 171 and $193~\mathring{\mathrm{A}}$ passbands.

### A.2 STEREO-A Coronagraph Results

We now repeated our automated fitting procedure described in Section 3.4, but fitting uniform (white-noise) confidence levels to 15-hour detrended data across the entire COR1 and COR2 fields of view. The smooth-subtracted AIA light curves in Figure 12 often yielded several peaks in Fourier power in the 6 – 13 hour range of periods (as seen in Section A.1). For the COR data, we recorded the Fourier power found in each pixel in two bins, between 6 – 9 hours and 9 – 13 hours, storing the maximum Fourier power in each bin above the confidence level.

In order to enhance any trends in Fourier power in the COR1 and COR2 fields of view, we present the ratio of the Fourier power in the longer (9 – 13-hour) to the shorter (6 – 9-hour) period bin as a map. Colours in the map represent the ratio value; negative values indicate that the magnitude of short-period FFT power is greater than the magnitude of longer-period FFT power, while positive values indicate that long-period FFT power is typically greater in magnitude than that in the shorter-period range. Colours are only assigned for FFT power values that are above the $95\%$ confidence interval in both period bins. Pixels with power that does not rise above the confidence level (in either bin) are presented in black. We present the maps for this power ratio in both the COR1 and COR2 fields of view in Figure 13.

Each power-map ratio in Figure 13 seems to reveal a significant amount of underlying structure (which is difficult to distinguish when simply plotting maps of the peak Fourier power in any one specific bin). The majority of structures appear to support more 9 – 13 hour oscillatory power than 6 – 9 hour FFT power. This is unsurprising as we noted an increase in FFT power with period in the original spectra. Furthermore, our earlier findings have suggested that “active” solar regions support spectra that are steeper than quiescent regions. Crucially, however, several regions also show more shorter-period FFT power dominating longer-period power. This is most readily apparent close to the Equator in Figure 13b, where the power ratio is greater than an order of magnitude, which directly contrasts with the overall increase in background Fourier power seen when moving from short to long periods throughout this investigation.

Three clear radial bands seen in red are visible in Figure 13a, indicating three latitudes where long-period oscillatory power dominates the shorter-period power to large heights. The strongest of these bands lies at approximately $55^{\circ}$. Another appears close to the occulter at $70^{\circ}$, but drifts towards $80^{\circ}$ latitude with height. A final band originates close to $80^{\circ}$ near the occulter and again drifts equator-ward with height. Several shorter or less intense features are also visible. The strongest and largest of the visible bands persist into the COR2 FOV in Figure 13b, and they are well aligned with latitudes at which various open structures were identified in STEREO/EUVI images in Figure 6 and in on-disk SDO/AIA images in Figure 1. The general shape of the radial bands is consistent with the large-scale magnetic structure of the corona at the time of the observations, as outlined in co-temporal C2 and C3 SOHO/LASCO images (not shown). Closer to the Sun, the bands become less distinct, likely as a result of the dynamic behaviour of the active regions close to the solar surface (with interference from more short-lived features). These features and periods match the ranges reported in the literature by, for example, Telloni *et al.* (2013).
